# A Repetitive Transcranial Magnetic Stimulation–Functional Near-Infrared Spectroscopy System: Achieving Dynamic Monitoring of Neuroplasticity in Clinical Rehabilitation

**DOI:** 10.34133/bmef.0283

**Published:** 2026-07-02

**Authors:** Hui Xie, Yan Wang, Xin Li, Zengyong Li, Lin Wang, Gongcheng Xu, Daifa Wang, Yinghu Peng, Zulin Dou, Qitao Tan, Ming Zhang

**Affiliations:** ^1^ Department of Biomedical Engineering, Faculty of Engineering, The Hong Kong Polytechnic University, Hong Kong SAR 999077, China.; ^2^ Research Institute for Sports Science and Technology, The Hong Kong Polytechnic University, Hong Kong SAR 999077, China.; ^3^ Department of Rehabilitation Medicine, The Third Affiliated Hospital of Sun Yat-sen University, Guangzhou 510630, China.; ^4^ Beijing Key Laboratory of Rehabilitation Technical Aids for Old-Age Disability, National Research Center for Rehabilitation Technical Aids, Beijing 100176, China.; ^5^ CAS Key Laboratory of Human-Machine Intelligence-Synergy Systems, Shenzhen Institutes of Advanced Technology, Chinese Academy of Sciences, Shenzhen 518055, China.; ^6^ School of Biological Science and Medical Engineering, Beihang University, Beijing 100191, China.

## Abstract

**Objective:** This study aimed to integrate repetitive transcranial magnetic stimulation (rTMS) with functional near-infrared spectroscopy (fNIRS) to achieve concurrent monitoring of neural activity during neuromodulation and to evaluate its feasibility in stroke rehabilitation. **Impact Statement:** This work addresses the long-standing lack of concurrent feedback in stroke neuromodulation and provides an important pathway for developing adaptive, closed-loop neurorehabilitation technologies. **Introduction:** The application and optimization of rTMS in stroke rehabilitation are constrained by the absence of concurrent physiological feedback, which limits timely evaluation of stimulation efficacy and adjustment of treatment parameters. Existing techniques combining neuromodulation with neuroimaging often lack validation, hindering their clinical translation and widespread application. **Methods:** This study employed improved fNIRS probes to construct an rTMS–fNIRS system capable of proximal detection under routine clinical rTMS conditions. Magnetic interference testing and simulation analyses were performed to evaluate system artifacts, field penetration, and focality. A 14-d clinical trial was conducted to verify the system’s feasibility and effectiveness. A total of 80 patients with stroke were enrolled, and wavelet amplitude and laterality index were used to quantify neuroplasticity changes while clinical scales were applied to assess behavioral improvements. **Results:** Engineering validation demonstrated that the system stably recorded cortical hemodynamic responses under high-intensity stimulation. Concurrent clinical monitoring revealed that excitatory rTMS induced dynamic enhancement of cortical activation and interhemispheric rebalancing, and these neurophysiological changes were significantly correlated with improvements in upper-limb motor recovery. **Conclusion:** The proposed rTMS–fNIRS system not only provides stable neuromodulatory intervention but also enables concurrent monitoring of stimulation-induced neuroplastic changes under clinical conditions.

## Introduction

Stroke is a leading cause of long-term disability worldwide, with approximately 80% of survivors exhibiting motor dysfunction, particularly in the upper limb [1]. Functional recovery after stroke relies heavily on cortical reorganization driven by neural plasticity [2]. Although conventional rehabilitation remains fundamental to post-stroke motor recovery, current rehabilitation practice still requires further optimization to better meet clinical needs and improve motor outcomes [3]. Accordingly, neuromodulation technologies, especially repetitive transcranial magnetic stimulation (rTMS), have attracted increasing attention as adjunctive strategies to modulate cortical excitability, promote neuroplasticity, and facilitate motor recovery [4,5].

Although rTMS is increasingly used in stroke rehabilitation, its efficacy is most often inferred indirectly from behavioral improvements that emerge only after prolonged neuroplasticity. This evaluation latency hampers timely verification of treatment effectiveness and parameter optimization, increasing the risk of suboptimal dosing, either under- or over-stimulation, and ultimately blunting therapeutic benefit [6,7]. A typical example is high-frequency rTMS (HF-rTMS) targeting the ipsilesional motor cortex, which holds strong theoretical and translational promise for restoring excitability in impaired pathways [8]. Nevertheless, clinical guidelines still rate rTMS for stroke as Level B evidence [9–12], reflecting heterogeneity in prior results [13], and this variability is further compounded by the absence of immediate physiological readouts during stimulation.

Integrating neuromodulation with neuroimaging can address this gap by providing physiological readouts during stimulation. Currently, the main neuroimaging modalities combined with rTMS include functional magnetic resonance imaging (fMRI), electroencephalography (EEG), and functional near-infrared spectroscopy (fNIRS). rTMS–fMRI provides high spatial resolution, but its combination with rTMS remains technically challenging because of electromagnetic compatibility requirements and limited portability [14]. rTMS–EEG offers millisecond-level temporal resolution and relatively low cost, but TMS-induced electromagnetic and physiological artifacts can substantially interfere with EEG recordings near the stimulation site [15]. In contrast, fNIRS is characterized by resistance to electromagnetic interference, moderate spatiotemporal resolution, and low susceptibility to motion artifacts [16], making it an ideal imaging modality to pair with rTMS under clinical conditions. However, existing rTMS–fNIRS efforts remain largely exploratory and often do not implement near-instantaneous synchronization during stimulation [17]. Moreover, studies claiming real-time assessment frequently lack rigorous engineering validation, including precise synchronization of stimulation and imaging signals, verification and mitigation of electromagnetic interference, analysis of magnetic-field attenuation, and demonstration of monitoring stability under active stimulation, limitations that hinder clinical translation [18].

Consequently, this study aimed to design and validate an integrated rTMS–fNIRS system capable of concurrent evaluation of neuromodulatory effects during rTMS intervention, with specific attention to electromagnetic-interference control and magnetic-field effectiveness. We then implemented this system in a randomized, sham-controlled clinical study to interrogate the neuromodulatory effects of HF-rTMS on cortical neuroplasticity and motor recovery in patients with stroke, thereby validating the system’s stability and efficacy in realistic clinical scenarios. The successful construction and validation of this system provide a mature, stable, and reliable engineering solution, laying a crucial foundation for future closed-loop neuromodulation strategies and paving the way for personalized, adaptive, real-time interventions in stroke rehabilitation.

## Results

### Engineering validation

#### Cortical magnetic field distribution

**Fig. 1.**
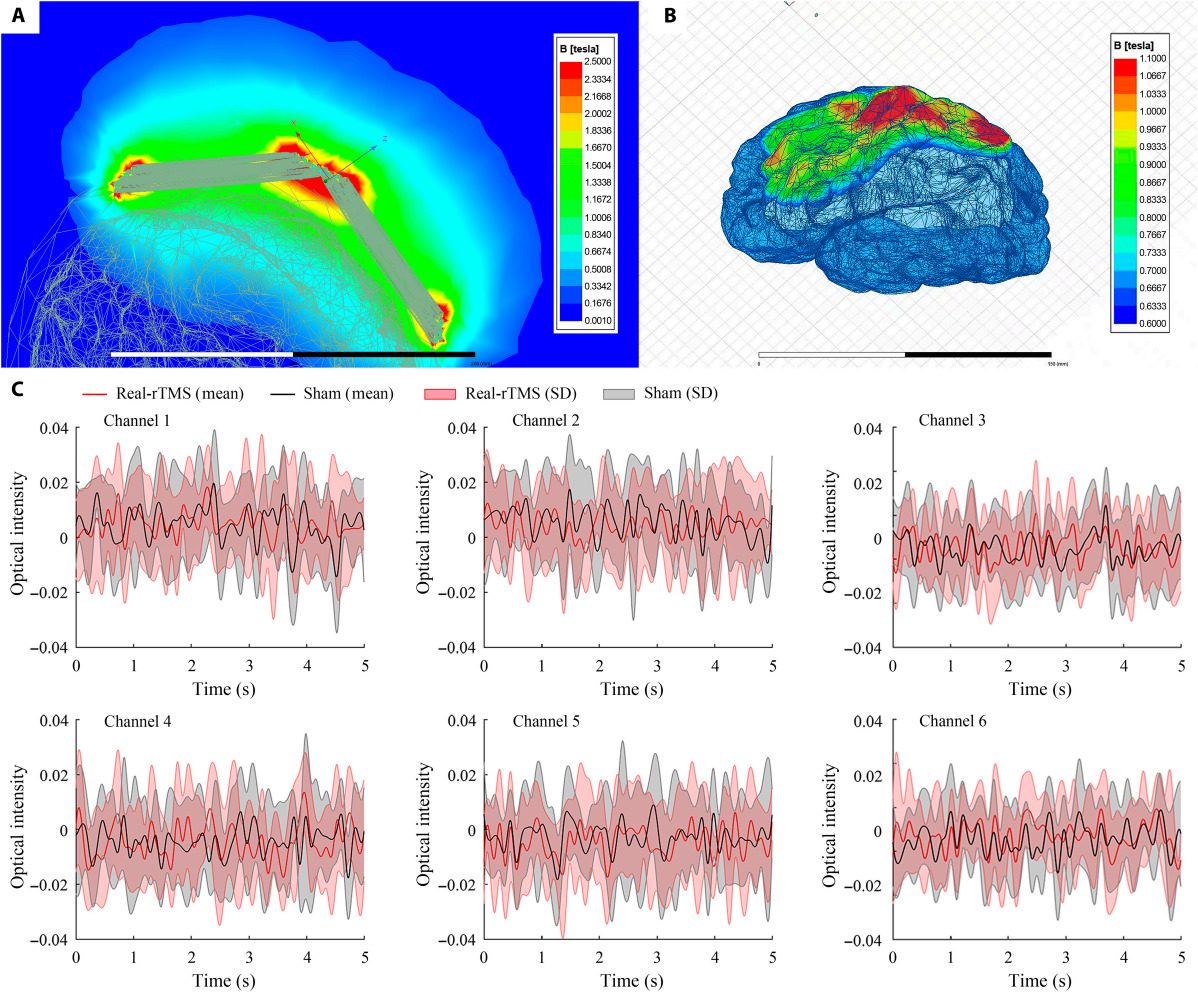
Engineering validation results. (A) Regional variations in magnetic-field intensity. (B) Cortical surface distribution of the magnetic field. The color scale represents the magnitude and range of field strength; warm colors indicate higher intensity, while cool colors indicate lower intensity. (C) Comparison of the magnetic field’s influence on functional near-infrared spectroscopy (fNIRS) light emission and detection between real repetitive transcranial magnetic stimulation (rTMS) and sham conditions in the rTMS–fNIRS system.

Simulation analysis was performed to characterize the cortical distribution of the effective magnetic field generated by the rTMS–fNIRS system (Fig. 1). In the realistic head model reconstructed from patient MRI scans, the magnetic-field source was positioned 25 mm above the targeted cortical surface with an initial peak intensity of 2.5 T. After traversing scalp and skull tissues, the induced magnetic field reached the cortical gray matter, with its spatial attenuation pattern illustrated in Fig. 1A. The simulation further revealed that the attenuated field maintained a peak intensity above 1 T at the cortical surface, with the highest values concentrated within a 30-mm-diameter region, indicating good focality (Fig. 1B).

#### Electromagnetic interference

The effects of the magnetic field on the rTMS–fNIRS system are shown in Fig. 1C. Optical intensity signals at 760 nm were compared across 6 independent detection channels under different conditions. The results indicated that during continuous excitatory rTMS intervention, the mean and standard deviation of both the detected optical signal intensity and its spectral distribution did not differ significantly from those observed under the sham condition.

### Clinical validation

#### Demographic characteristics

During the study period, a total of 118 patients with stroke were hospitalized for rehabilitation treatment. Among them, 12 did not meet the inclusion criteria, 16 were excluded, and 10 declined to participate, leaving 80 patients enrolled in the study. During the experiment, 8 participants were excluded from the final analysis due to probe loosening and 24 participants were excluded due to active withdrawal. The remaining 48 participants were included the data analyses, including sham (*n* = 23) and HF-rTMS (*n* = 25). Baseline demographic and clinical characteristics were comparable between the 2 groups. No significant differences were found in demographic characteristics, stroke characteristics, or baseline clinical scores, including Fugl–Meyer Assessment of the Upper Extremity (FMA-UE), Action Research Arm Test (ARAT), Modified Barthel Index (mBI), National Institutes of Health Stroke Scale (NIHSS), and Modified Rankin Scale (mRS), as shown in Table 1.

**Table 1.** Comparison of the baseline characteristics of the participants

Characteristics	Sham (*n* = 23)	HF-rTMS (*n* = 25)	*P* value
Gender (male/female)	19/4	19/6	0.728
Age (y)	58.37 ± 6.78	59.42 ± 8.21	0.871
Type (infarction/hemorrhage)	14/9	18/7	0.543
Ipsilesional hemisphere (right/left)	13/10	10/15	0.386
Post-onset duration (d ± SD)	64.96 ± 44.39	56.42 ± 38.16	0.452
Behavioral measures (score ± SD)			
FMA-UE	21.13 ± 16.34	24.04 ± 20.85	0.595
NIHSS	5.83 ± 2.62	5.72 ± 2.88	0.895
mBI	57.70 ± 26.84	53.28 ± 26.72	0.571
ARAT	8.39 ± 14.45	10.88 ± 17.19	0.591
mRS	3.52 ± 0.73	3.52 ± 0.87	0.994

HF-rTMS, high-frequency repetitive transcranial magnetic stimulation; FMA-UE, Fugl–Meyer Assessment of the Upper Extremity; ARAT, Action Research Arm Test; mBI, Modified Barthel Index; NIHSS, National Institutes of Health Stroke Scale; mRS, modified Rankin Scale

#### Primary outcome measures

The clinical outcomes of upper-limb motor function, stroke severity, activities of daily living, fine motor performance, and global functional independence are summarized in Table 2. Following the 14-d intervention, both the HF-rTMS and sham groups showed within-group improvements across all clinical scales, although none of these changes reached statistical significance.

**Table 2.** Differences between and within groups in primary outcome measure: mean ± SD

Scale	Group	Pre	Post	Within-group *P* value	14-d change	Between-group *P* value
FMA-UE	Sham	21.13 ± 16.34	24.78 ± 16.64	0.457	3.65 ± 2.21	0.027*
	HF-rTMS	24.04 ± 20.86	29.52 ± 21.14	0.361	5.96 ± 4.35	
NIHSS	Sham	5.83 ± 2.62	4.87 ± 2.82	0.241	−0.96 ± 1.07	0.111
	HF-rTMS	5.72 ± 2.88	4.32 ± 2.73	0.084	−1.40 ± 0.82	
mBI	Sham	57.70 ± 26.84	64.22 ± 26.19	0.409	6.52 ± 8.83	0.170
	HF-rTMS	53.28 ± 26.72	63.68 ± 23.30	0.149	10.40 ± 10.32	
ARAT	Sham	8.39 ± 14.45	11.65 ± 18.08	0.503	3.26 ± 9.08	0.756
	HF-rTMS	10.88 ± 17.19	13.52 ± 18.19	0.600	2.64 ± 3.93	
mRS	Sham	3.52 ± 0.73	3.13 ± 0.76	0.081	−0.39 ± 0.50	0.956
	HF-rTMS	3.52 ± 0.87	3.12 ± 0.83	0.104	−0.40 ± 0.58	

* *P* < 0.05.

The HF-rTMS group demonstrated a significantly greater improvement in FMA-UE score compared to the sham group. Specifically, the change in FMA-UE score was significantly higher in the HF-rTMS group than in the sham group (*P* = 0.027). Changes in the NIHSS, ARAT, mBI, and mRS scores were comparable between groups, with no statistically significant between-group differences. Taken together, the primary behavioral difference between groups was observed in the FMA-UE scores.

#### Secondary outcome measures

To explore intervention-associated changes in neurovascular coupling while accounting for inter-individual baseline variability, a baseline-adjusted region of interest (ROI)-level analysis of covariance (ANCOVA) was conducted for the fNIRS-derived secondary outcome. For each ROI, the value at T1 or T2 was entered as the dependent variable, group was entered as the between-subject factor, and the corresponding baseline value was included as a covariate.

**Fig. 2.**
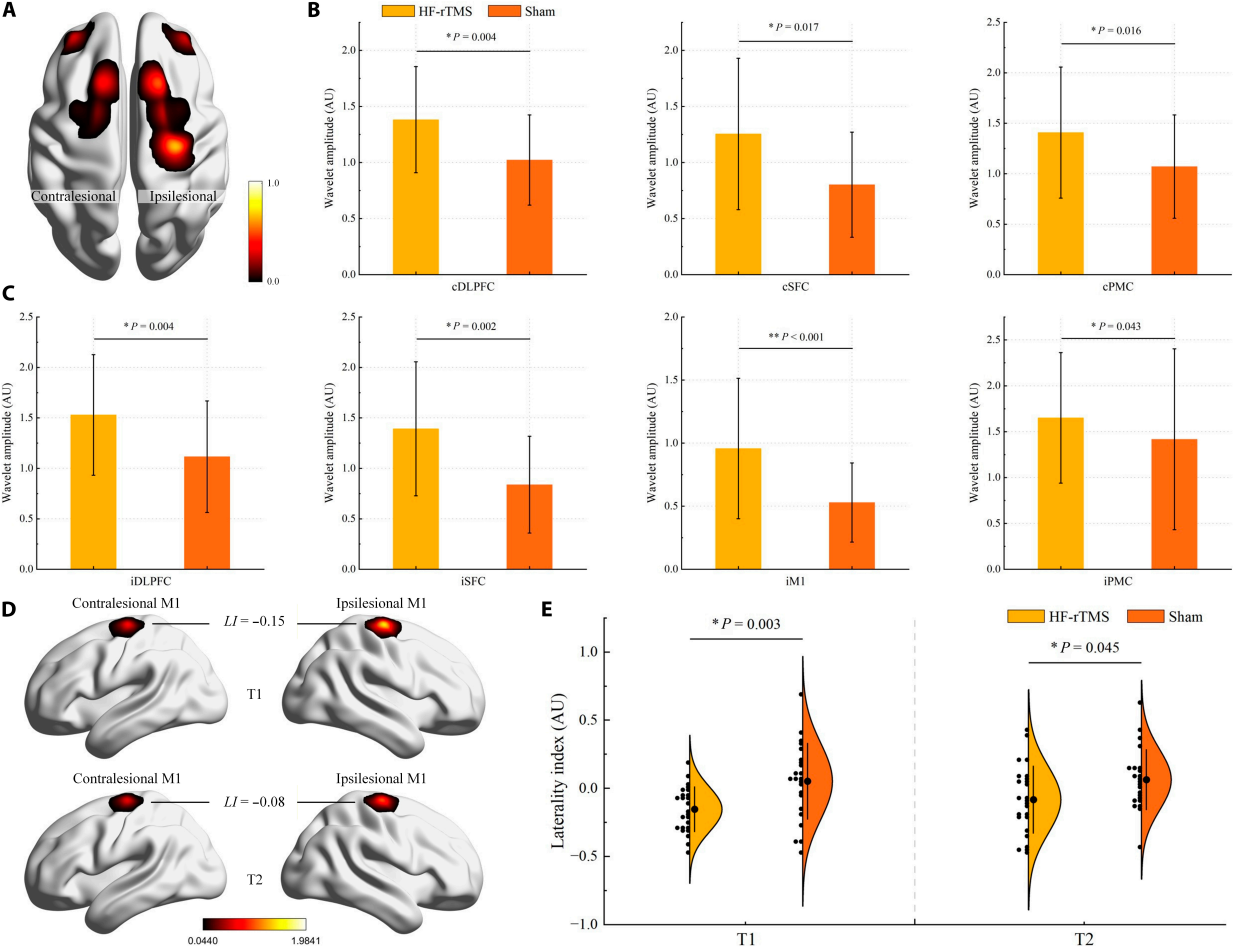
Significant differences in secondary outcome measures between the HF-rTMS and sham groups. (A) Spatial distribution of regions with significant between-group WA differences at T1. (B and C) Region of interest (ROI)-level comparisons of wavelet amplitude (WA) in significant contralesional (c) and ipsilesional (i) regions at T1. (D) Spatial distribution of regions with significant between-group laterality index (LI) differences at T1 and T2. (E) ROI-level comparisons of LI between the HF-rTMS and sham groups at T1 and T2. DLPFC, dorsolateral prefrontal cortex; SFC, superior frontal cortex; PMC, premotor cortex.

As an rTMS–fNIRS-derived indicator, wavelet amplitude (WA) was used to characterize cortical activation. At T1, after adjustment for baseline WA, significantly higher WA values were observed in the HF-rTMS group than in the sham group across several ROIs, as shown in Fig. 2A. Specifically, in the contralesional hemisphere (c), significant between-group differences were found in the dorsolateral prefrontal cortex (cDLPFC, *F* = 9.408, *P* = 0.004, *ηp*^2^ = 0.173), superior frontal cortex (cSFC, *F* = 6.171, *P* = 0.017, *ηp*^2^ = 0.121), and premotor cortex (cPMC, *F* = 6.328, *P* = 0.016, *ηp*^2^ = 0.123), as shown in Fig. 2B. In the ipsilesional hemisphere (i), significant differences were also observed in iDLPFC (*F* = 9.508, *P* = 0.004, *ηp*^2^ = 0.174), iSFC (*F* = 10.978, *P* = 0.002, *ηp*^2^ = 0.196), primary motor cortex (iM1, *F* = 16.023, *P* < 0.001, *ηp*^2^ = 0.263), and iPMC (*F* = 4.360, *P* = 0.043, *ηp*^2^ = 0.088), as shown in Fig. 2C. At T2, no significant baseline-adjusted between-group differences were observed in any ROI.

Changes in cortical laterality were also analyzed using the laterality index (LI). Figure 2D shows the regions with significant between-group differences in LI between the HF-rTMS and sham groups at T1 and T2. At T1, after adjustment for baseline LI, the LI value in M1 was significantly lower in the HF-rTMS group than in the sham group (*F* = 9.745, *P* = 0.003, *ηp*^2^ = 0.178). At T2, a similar baseline-adjusted between-group difference was also observed in M1 (*F* = 4.271, *P* = 0.045, *ηp*^2^ = 0.087), as shown in Fig. 2E. No significant differences were observed in the other ROIs.

#### Correlation between rTMS–fNIRS-derived neurophysiological indicators and motor recovery

**Fig. 3.**
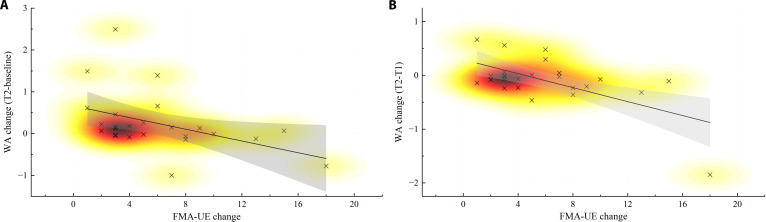
Relationships between motor recovery and cortical response changes induced by the rTMS–fNIRS system. Scatter points represent individual patients and the red–yellow density maps indicate the concentration of data points. (A) WA change from T2 to baseline was negatively correlated with ΔFMA-UE. (B) WA change from T2 to T1 also showed a significant negative correlation with ΔFMA-UE. FMA-UE, Fugl–Meyer Assessment of the Upper Extremity.

To explore the relationship between intervention-induced clinical motor recovery and neurovascular coupling changes, we extracted significant findings from both primary and secondary outcome measures. Pearson correlation analyses were performed in the HF-rTMS group to assess the association between the 14-d improvement in upper-limb motor function (ΔFMA-UE) and changes in fNIRS-derived indicators across 3 time intervals: from baseline to T1 (T1-baseline), from baseline to T2 (T2-baseline), and from T1 to T2 (T2-T1) (Fig. 3). Among the analyzed cortical regions, significant negative correlations were observed between ΔFMA-UE and ΔWA in the iM1. Specifically, ΔWA (T2-baseline) showed a negative correlation with ΔFMA-UE (*r* = −0.471, *P* = 0.018) (Fig. 3A), and ΔWA (T2-T1) also demonstrated a significant negative correlation (*r* = −0.615, *P* = 0.001) (Fig. 3B).

## Discussion

This study designed and validated an integrated rTMS–fNIRS system capable of delivering neuromodulatory stimulation while concurrently monitoring cortical hemodynamic responses. Engineering validation showed that the customized nonmetallic optode configuration and silicone padding enabled stable optical signal acquisition under active rTMS, and simulation analysis confirmed sufficient cortical magnetic-field penetration and acceptable spatial focality under clinically relevant conditions. In the clinical validation, the integrated rTMS–fNIRS system demonstrated its practical value as both a neuromodulatory intervention platform and a concurrent monitoring platform. The system promoted upper-limb motor recovery in patients with stroke, while also reliably extracting concurrent, noninvasive hemodynamic biomarkers during the intervention; these neurophysiological changes were closely associated with functional recovery. Overall, these results indicate that the proposed system has extended beyond technical proof of concept toward clinical validation and lays the foundation for the future development of closed-loop neuromodulation.

### rTMS–fNIRS system validation

This study conducted magnetic-field interference tests and simulation analyses that provide concrete evidence for the effectiveness of the integrated rTMS–fNIRS system. First, electromagnetic-interference testing showed that continuous, high-intensity active rTMS did not significantly alter optical signal quality, indicating that the system can stably acquire hemodynamic signals during operation. This finding is particularly important because prior studies have identified electromagnetic artifacts and coil-movement artifacts as major barriers to the practical integration of rTMS and fNIRS. On the one hand, any internal component of the optical instrument may generate electromagnetic interference due to changes in the magnetic field; on the other hand, mechanical contraction of the rTMS coil can cause subtle displacement of the optodes, thereby affecting the optical signal. To minimize coil-movement interference, we introduced a small spatial gap between the coil and the optodes to avoid physical contact; in addition, customized nonmetallic optode housings and silicone padding contributed substantially to suppressing these artifacts.

Second, because the magnetic field produced by a TMS coil decays rapidly with distance, adding only a few millimeters can markedly reduce stimulation efficacy for the cortex and subcortical regions. Previous work often adopted coil-surround optical layouts paired to specific TMS coils, placing optodes around the coil edge or aperture [19], which minimizes the coil–target distance but constrains channel configuration for recording ROIs. Accordingly, we redesigned the rTMS–fNIRS layout so that the optode array could be positioned directly beneath the coil. As this geometry inevitably introduces additional coil–cortex distance, we employed customized 10-mm optodes to reduce spacing; nevertheless, attenuation of field strength with distance must be accounted for to ensure sufficient stimulation of the target.

The magnetic-field strength reaching the cortical target and its spatial focality are core indices for evaluating the effectiveness of rTMS intervention; validating both is therefore critical for assessing our system. Because magnetic fields within the living head are difficult to measure directly, we performed finite-element modeling, constructing both the cranial tissues and the coil. Unlike many prior approaches that relied on simplified spherical head models, we reconstructed a patient-specific 3-dimensional head model from MRI and conducted finite-element simulations to approximate realistic anatomy and conductivity boundaries [20]. The simulations indicated that, under clinically realistic configurations, peak magnetic flux density near the cortical surface reached magnitudes consistent with conventional coils (≥1 T). Prior studies report peak magnetic flux densities of approximately 1 to 2 T near the coil face; when combined with appropriate temporal rates of change, such fields are sufficient to reach motor threshold in the cortex. Moreover, the distribution of the main high-field region in the simulations demonstrated the system’s focality, and its full width at half maximum and equivalent diameter conformed to the canonical depth–focality trade-off [21]. Taken together, these results suggest that the proposed rTMS–fNIRS system meets clinical requirements for penetration depth and spatial focality, enabling effective stimulation of the target while limiting nonspecific effects in nontarget regions.

### The rTMS–fNIRS system detected neurophysiological effects

The system detected significantly higher WA in the HF-rTMS group than in the sham group at T1 across bilateral cortical regions. This finding indicates that the system was sensitive enough to capture early cortical activation induced by HF-rTMS. The involvement of iM1 and iPMC suggests engagement of the ipsilesional motor network, whereas activation in DLPFC and SFC may reflect bilateral cortical activation during early neuromodulatory adaptation [22]. This pattern is consistent with previous neuroimaging evidence showing that rTMS can modulate both motor and nonmotor cortical regions involved in post-stroke functional recovery [23]. Therefore, these WA findings support the value of the rTMS–fNIRS system for monitoring stimulation-related cortical recruitment beyond the local stimulation target.

Notably, no significant baseline-adjusted between-group differences in WA were observed at T2. This result should not be interpreted simply as the absence of a neurophysiological effect. Instead, when considered together with the significant T1 activation pattern, it may indicate that the hemodynamic response to HF-rTMS is dynamic rather than linearly increasing over time. The early enhancement of WA may represent acute cortical recruitment or stimulation responsiveness, whereas the absence of persistent WA elevation at T2 may reflect response normalization, reduced hemodynamic demand, or improved neural efficiency after repeated stimulation and rehabilitation training. However, nonspecific rehabilitation-related effects in both groups cannot be fully excluded [24,25]. This temporal pattern further suggests that the neurophysiological effect of HF-rTMS is not a simple linear increase in cortical activation but a dynamic process involving early recruitment followed by later adaptation or normalization.

The LI results further indicate that, during the intervention, the rTMS–fNIRS system delivered HF-rTMS capable of promoting hemispheric reorganization and simultaneously captured the resulting changes in hemispheric balance. The lower M1 LI in the HF-rTMS group suggests that stimulation delivered through the integrated system promoted iM1 dominance. This finding is consistent with the therapeutic rationale of applying excitatory rTMS to the lesioned hemisphere to enhance ipsilesional cortical excitability and support motor network reorganization after stroke [26]. In contrast, the relatively higher LI in the sham group may indicate greater cM1 dominance, which could reflect compensatory reliance on the unaffected hemisphere and, if sustained, may be associated with increased interhemispheric imbalance and less optimal motor recovery [27,28]. Together, these findings suggest that HF-rTMS primarily influenced M1 hemispheric rebalancing, rather than inducing a nonspecific global shift in cortical lateralization.

### The rTMS–fNIRS system induced clinical behavioral changes

In addition to neurophysiological effects, this study demonstrated clinical behavioral improvements induced by the rTMS–fNIRS system. Although not all clinical outcome measures reached statistical significance, the observed within-group trends offer important insights into the potential therapeutic efficacy of both interventions.

In the HF-rTMS group, numerical improvements were detected in both the NIHSS and mRS scores over the 14-d intervention period, while the sham group similarly demonstrated a trend toward improved mRS scores. These findings suggest that both conventional rehabilitation and integrated rTMS–fNIRS intervention can positively influence global neurological function and functional independence within a relatively short time frame. However, the short intervention duration may have limited the statistical detectability of behavioral effects at the group level. This underscores the potential need for more sensitive clinical or neuroimaging tools to fully capture the impact of neuromodulation during the subacute phase of stroke recovery.

Crucially, between-group comparisons revealed a statistically significant improvement in FMA-UE scores in the HF-rTMS group compared to the sham group. This finding provides direct clinical evidence that the integrated rTMS–fNIRS system, despite the modified spatial configuration between the coil and cortex, can still deliver effective neuromodulatory stimulation and produce meaningful motor recovery in practice.

Collectively, the significant FMA-UE improvement and favorable trends in other clinical outcomes confirm that the rTMS–fNIRS system is not only technically feasible but also clinically effective. These results demonstrate that the system can deliver therapeutic stimulation in real rehabilitation scenarios, thereby validating its practical applicability beyond theoretical modeling.

### Correlation between neurophysiological responses and motor recovery

Building on the behavioral findings, we further examined whether neurophysiological responses detected by the rTMS–fNIRS system were associated with upper-limb motor recovery. The change from baseline to T2 reflects the overall alteration in iM1 hemodynamic response after the full 14-d intervention, whereas the change from T1 to T2 reflects the evolution from the initial stimulation response to the response observed at the end of repeated intervention.

These 2 negative correlations point to a consistent pattern: Patients with greater motor recovery tended to show lower iM1 WA at T2 relative to both their baseline state and their early-stimulation response. Therefore, better recovery was not associated with sustained iM1 hyperactivation. Instead, it may reflect a transition from early-stimulation-induced cortical recruitment to later normalization or reduced hemodynamic demand within the ipsilesional motor cortex [29,30].

This interpretation is also consistent with the group-level WA results. The HF-rTMS group showed higher WA than the sham group at T1, indicating early cortical recruitment induced by stimulation [31], whereas no significant between-group WA differences were observed at T2. Together with the negative correlations between iM1 WA changes and FMA-UE improvement, these findings suggest that the neurophysiological response to HF-rTMS is dynamic rather than persistently enhanced. Early WA elevation may reflect target engagement and cortical responsiveness, while the subsequent reduction or normalization of iM1 WA may indicate adaptive recalibration, improved neural efficiency, or homeostatic regulation after repeated stimulation and rehabilitation [32]. Such a transition from early recruitment to later normalization may be associated with more favorable motor recovery [33,34].

### Limitations

This study has several limitations. First, the current rTMS–fNIRS configuration did not include short separation channels. Conventional short separation designs require additional source detector pairs placed close to the scalp surface and are also constrained by the material compatibility of electronic and optical components. Such configurations are difficult to implement beneath an rTMS coil and may reduce the effective magnetic field reaching the cortical target. To mitigate this limitation, we used independent-component-analysis- and principal-component-analysis-based preprocessing to reduce components related to scalp blood flow, respiratory and cardiac activity, and motion artifacts. However, these methods cannot fully replace direct short separation regression. Future optimization of the system should focus on developing rTMS-compatible short separation probes, so that cortical and extracerebral signals can be better separated without compromising stimulation efficiency. Second, the finite-element simulation was intended to validate the engineering feasibility of the integrated configuration, rather than to guide stimulation dose allocation. Future studies should combine participant-specific MRI-based magnetic-field modeling with concurrent fNIRS feedback to further optimize individualized neuromodulation strategies.

## Conclusion

This study designed and validated an rTMS–fNIRS system capable of delivering neuromodulatory intervention while concurrently monitoring stimulation-induced cortical hemodynamic responses. Magnetic-field interference testing and simulation analyses confirmed the technical feasibility of the system. Clinical validation further demonstrated stable hemodynamic monitoring capability and a selective therapeutic benefit for upper-limb motor recovery in patients with subacute stroke. The observed associations between dynamic iM1 hemodynamic changes and FMA-UE improvement provide physiological evidence for evaluating individual responsiveness to HF-rTMS. These findings support the potential of the rTMS–fNIRS system as a translational platform for feedback-guided and individualized neuromodulation in stroke rehabilitation.

## Materials and Methods

### Integrated rTMS–fNIRS system design

**Fig. 4.**
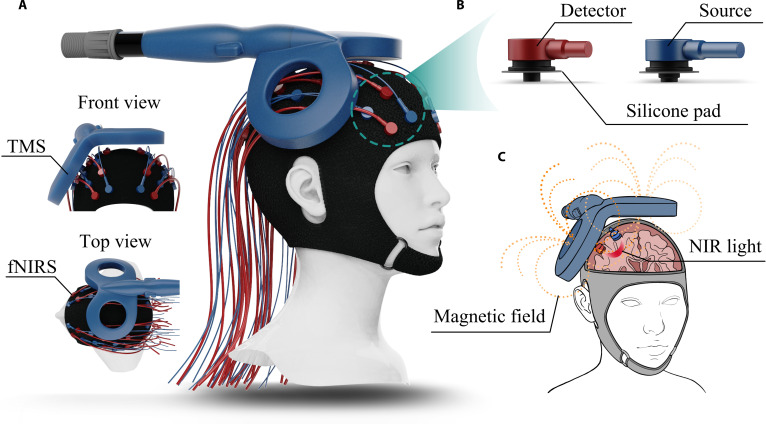
Configuration of the integrated rTMS–fNIRS system. (A) Main system components, including the engineering overview together with front and top views. (B) Customized optode layout illustrating the distribution of light sources and detectors, each encapsulated in a 10-mm housing and equipped with silicone padding to minimize distance-related magnetic-field attenuation and motion artifacts. (C) Schematic of the overall operating principle, illustrating that the rTMS stimulation target and fNIRS detection area are co-localized, thereby enabling effective monitoring.

The integrated rTMS–fNIRS system consisted of a commercial rTMS stimulator (NS1000; Yiruide, Wuhan, China) equipped with a butterfly coil and a continuous-wave fNIRS imaging device (NirSmart; Huichuang Medical Equipment Co., Beijing, China), enabling the coupling of neuromodulation with neuroimaging (Fig. 4A). Precise synchronization between stimulation and imaging was achieved through transistor–transistor logic signaling. At the onset of the first rTMS pulse, the experimental control computer generated a transistor–transistor logic trigger that was simultaneously embedded in the fNIRS data stream. This event marker allowed millisecond-level temporal alignment of rTMS delivery with fNIRS acquisition, thereby ensuring that stimulation events were accurately mapped onto the corresponding hemodynamic responses.

To minimize electromagnetic interference from the rTMS coil, customized fNIRS sources and detectors were employed. These probes featured a flat 10-mm housing with internal optical fibers and no metallic components, reducing the likelihood of induction artifacts around the detectors. To further suppress motion-related artifacts caused by head vibration during stimulation, the rTMS coil was carefully positioned above the fNIRS probe array, avoiding direct or indirect physical contact, and a flexible silicone pad was placed around the fNIRS probes (Fig. 4B). These engineering measures ensured reliable acquisition of hemodynamic signals during stimulation. The overall operating principle of the integrated system, combining magnetic-field induction and infrared-light propagation, is illustrated in Fig. 4C.

### Engineering validation

This study conducted engineering validation of the integrated rTMS–fNIRS system by systematically examining electromagnetic interference and cortical magnetic-field distribution [35] in order to ensure its technical reliability and suitability for clinical application. Electromagnetic-interference testing was performed by acquiring and comparing fNIRS signals under 2 conditions: active rTMS and sham stimulation. For each condition, 10 trials were recorded, and the raw optical intensity at 760 nm from fNIRS channels was analyzed to assess whether the magnetic field generated by rTMS influenced signal quality. It should be noted that this MRI-based simulation was performed as an engineering validation of the integrated rTMS–fNIRS configuration, rather than as individualized neuronavigation for clinical coil placement. The aim was to determine whether the modified spatial arrangement of the coil, fNIRS probes, and cortex could still provide sufficient cortical magnetic-field penetration and acceptable focality before clinical application.

**Fig. 5.**
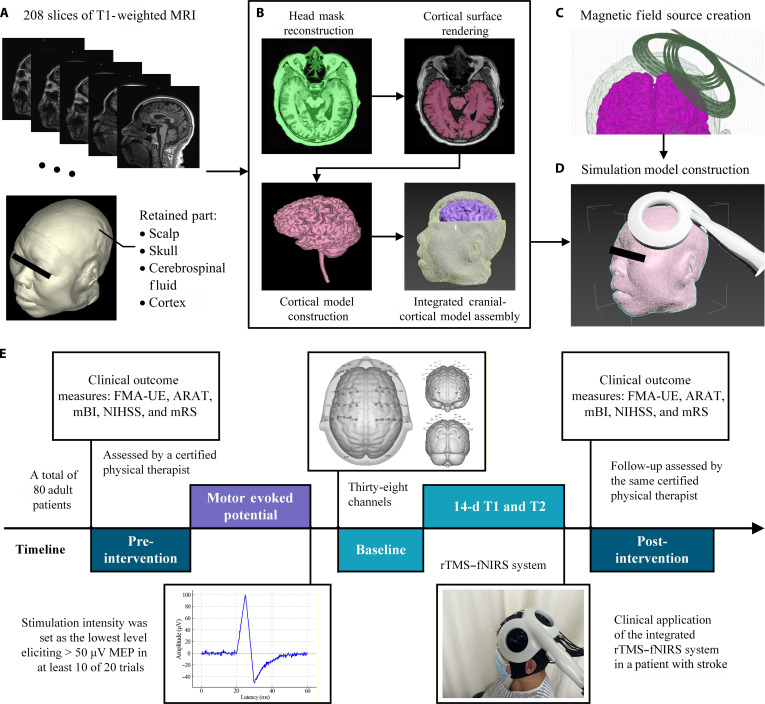
Simulation modeling and clinical validation workflow. (A) Three-dimensional head model reconstructed from T1-weighted MRI data of a patient with stroke. (B) Cranial model with an independent cortical layer obtained through tissue segmentation. (C) Construction of an external TMS magnetic-field model. (D) Layout of system components under clinical application conditions. (E) Clinical validation design and workflow. MEP, motor evoked potential; FMA-UE, Fugl–Meyer Assessment of the Upper Extremity; ARAT, Action Research Arm Test; mBI, Modified Barthel Index; NIHSS, National Institutes of Health Stroke Scale; mRS, modified Rankin Scale.

The cortical-level magnetic-field distribution of the rTMS coil was evaluated through simulation analysis. The construction process of the simulation model is illustrated in Fig. 5. A total of 208 high-resolution T1-weighted MRI slices from a patient with stroke were acquired to reconstruct a 3D head model, including scalp, skull, cerebrospinal fluid, and cortical gray matter (Fig. 5A). Tissue segmentation was applied to generate an integrated cranial–cortical model (Fig. 5B). A magnetic-field source was then constructed using the empirically measured maximum field strength of 2.5 T obtained with a teslameter (Fig. 5C). Finally, all components were assembled according to clinical configurations to establish the complete simulation model (Fig. 5D), which was subsequently analyzed to estimate magnetic-field distribution within the target region.

### Clinical validation

#### Participants

This study was designed as a double-blind, randomized, sham-controlled trial, and the overall study design is illustrated in Fig. 5E. A total of 80 adult patients (aged 20 to 80 y) with first-ever stroke and upper-limb motor impairment were recruited from the inpatient Department of Rehabilitation Medicine, The Third Affiliated Hospital of Sun Yat-sen University. Eligible participants were randomly assigned to either the rTMS group or sham group. Randomization was generated in MATLAB, and natural numbers from 1 to 80 were divided into 2 groups and sealed in sequentially numbered, opaque envelopes. The operator was unblinded but was not involved in outcome assessment or data analysis; participants and assessors remained blinded throughout the trial.

Sample size was calculated using G*Power 3.1 with an alpha level of 0.05, a power of 0.80, and an effect size of 0.25, indicating a minimum of 17 patients per group. Considering a 20% dropout rate, 22 participants were recruited for each group. Inclusion criteria were (a) subacute stroke onset (7 d to 6 mo), (b) right-handedness, (c) ability to follow simple instructions (Mini-Mental State Examination score > 21), and (d) residual unilateral upper-limb motor deficits. Exclusion criteria included (a) history of epilepsy or brain tumor; (b) severe comorbid cardiac, pulmonary, or renal disease; (c) contraindications for rTMS; and (iv ) infectious skin disease.

All participants provided written informed consent. The study was approved by the Human Ethics Committee of The Third Affiliated Hospital of Sun Yat-sen University ([2021] 02-333-01) and registered at the Chinese Clinical Trial Registry (ChiCTR2100054527).

#### Experimental design

Both groups received standard rehabilitation therapy individually tailored by physical therapists, including passive joint mobilization, muscle strengthening, and stretching exercises. In addition to standard rehabilitation, both groups underwent one daily rTMS session for 14 consecutive days, with stimulation targeted over the iM1. The intervention parameters were identical in setup, but real stimulation was applied in the rTMS group, whereas the sham group received placebo stimulation under the same procedural conditions.

The primary clinical outcome measures were changes in clinical scale scores. Clinical assessments were conducted before and after the 14-d intervention using the following validated scales: the FMA-UE for upper-limb motor function, the ARAT for fine motor control and dexterity, the mBI for activities of daily living, the NIHSS for global neurological impairment, and the mRS for overall functional independence. These outcomes were used to evaluate and compare the clinical efficacy of the 2 intervention protocols.

The secondary outcome measures were changes in neuroplasticity. All participants underwent concurrent rTMS–fNIRS assessments during resting state (baseline), during the initial intervention (T1), and during the final intervention on day 14 (T2). These measures were used to evaluate cortical hemodynamic responses and neuroplasticity-related changes induced by the intervention.

#### rTMS intervention

HF-rTMS was administered over the ipsilesional M1. Because M1 has a clear corticospinal output, the clinical stimulation target was functionally localized rather than determined by the simulated magnetic-field coordinates. The motor hotspot for the abductor pollicis brevis muscle on the ipsilesional M1 was identified using motor-evoked-potential-based localization, and the resting motor threshold (rMT) was determined as the minimum stimulation intensity required to elicit at least 10 motor evoked potentials of >50 μV in amplitude in 20 trials. If the rMT could not be elicited from the ipsilesional hemisphere, the homologous region in the contralesional hemisphere was used as a reference. HF-rTMS was delivered at 5 Hz with 2-s trains and 4-s inter-train intervals, for a total of 1,000 pulses at 100% rMT [36]. For sham stimulation, the coil was held at a 90-degree angle away from the scalp, reproducing similar auditory and somatosensory input without effective stimulation.

#### fNIRS data acquisition

At baseline, T1, and T2 time points, cortical hemodynamics were continuously recorded during intervention using the rTMS–fNIRS system. Based on the international 10-20 electrode system, 38 channels with 30-mm distance covered the ipsilesional and contralesional PFC, DLPFC, SFC, PMC, M1, S1, and occipital cortex. Before measurements, participants were instructed to sit quietly for 5 to 10 min to eliminate potential hemodynamic changes. We set all differential path-length factors to 7.0, and the sampling frequency was set at 10 Hz.

#### Data preprocessing and neural plasticity indicators

Raw optical density signals were band-pass filtered at 0.0095 to 2 Hz using a zero-phase fifth-order Butterworth filter to remove uncorrelated noise and low-frequency drift [37]. The filtered signals were converted to changes in oxygenated hemoglobin (Δ[oxy-Hb]) using the modified Beer–Lambert law [38]. To reduce noise artifacts, a combination of sliding average smoothing and cubic spline interpolation was applied. Further preprocessing involved independent component analysis and principal component analysis to remove physiological noise such as cardiac and respiratory signals, scalp blood flow, and motion-related interference [39]. All components were then visually inspected, and those exhibiting artifact-related features were excluded to enhance signal quality and improve the signal-to-noise ratio in the rTMS–fNIRS analysis.

To isolate neural-related hemodynamic activity, we applied a continuous wavelet transform using complex Morlet wavelets to extract oscillatory components in the 0.01 to 0.08 Hz frequency range, which primarily reflect spontaneous or task-evoked cortical activation. At a given frequency 𝑓 and time point 𝑇_𝑛_, the complex wavelet coefficient is defined as

$$\begin{array}{c} w_{k}\left(T_{n}\right)=W_{k}\left(f,\;T_{n}\right)\cdot e^{i\emptyset_{k}\left(f,T_{n}\right)}=a_{k}\left(f,\;T_{n}\right)+{\mathrm{ib}}_{k}\left(f,\;T_{n}\right) \end{array}$$ (1)

where 𝑎_𝑘_ and 𝑏_𝑘_ are the real and imaginary parts of the signal, respectively.

The wavelet transform results were averaged in the time domain to obtain WA values, representing the magnitude of regional cerebral blood flow fluctuations associated with neural activation, and were derived as

$${\mathrm{WA}}_{k}\left(f,\;T_{n}\right)=\sqrt{a_{k}^{2}\left(f,\;T_{n}\right)+b_{k}^{2}\left(f,\;T_{n}\right)}$$ (2)

Time-averaged WA values were computed for each ROI. These values were subsequently used to calculate the LI, which quantifies interhemispheric dominance by comparing activation between the ipsilesional and contralesional hemispheres [40]:

$$\mathrm{LI}=\frac{\left(\sum {\mathrm{WA}}_{\text{contralesional}}-\sum {\mathrm{WA}}_{\text{ipsilesional}}\right)}{\left(\sum {\mathrm{WA}}_{\text{contralesional}}+\sum {\mathrm{WA}}_{\text{ipsilesional}}\right)}$$ (3)

Here, ${\mathrm{WA}}_{\text{contralesional}}$ and ${\mathrm{WA}}_{\text{ipsilesional}}$ refer to the wavelet amplitudes from the contralesional and ipsilesional hemispheres, respectively. LI values range from +1 (completely contralesional activation) to −1 (completely ipsilesional activation), thereby serving as a measure of hemispheric asymmetry in cortical engagement.

#### Statistical analysis

The Kolmogorov–Smirnov test was used to assess the normality of data distributions, and Levene’s test was employed to evaluate the homogeneity of variance across groups. Independent sample *t* tests were used to compare the demographic characteristics and changes in clinical scales between the 2 groups of patients.

To account for baseline variability in fNIRS-derived indicators, baseline-adjusted ROI-level ANCOVA was performed for secondary outcome measures. For each ROI, the WA or LI value at T1 or T2 was entered as the dependent variable, group was entered as the between-subject factor, and the corresponding baseline value was included as a covariate. To reduce reliance on parametric assumptions, nonparametric permutation testing with 5,000 random permutations of group labels was further applied to the ROI-level ANCOVA. To explore the association between fNIRS-derived indicators and upper-limb motor recovery, Pearson correlation analysis was conducted between significantly altered ROI-level fNIRS indicators and changes in FMA-UE scores after the 14-d intervention. Bonferroni correction was applied to ANCOVA and Pearson correlation analyses. All statistical tests were 2 tailed, and a corrected *P* value <0.05 was considered statistically significant where applicable.

## Data Availability

The datasets generated during and/or analyzed during the current study are available from the corresponding author on reasonable request.
